# Identification of *TaPPH-7A* haplotypes and development of a molecular marker associated with important agronomic traits in common wheat

**DOI:** 10.1186/s12870-019-1901-0

**Published:** 2019-07-08

**Authors:** Huiyan Wang, Shuguang Wang, Xiaoping Chang, Chenyang Hao, Daizhen Sun, Ruilian Jing

**Affiliations:** 1 College of Agronomy, Shanxi Agricultural University, Taigu, 030801 China; 2 National Key Facility for Crop Gene Resources and Genetic Improvement/Institute of Crop Sciences, Chinese Academy of Agricultural Sciences, Beijing, 100081 China

**Keywords:** Gene cloning, Allelic variation, Molecular marker, TGW, Chl content, Wheat

## Abstract

**Background:**

Premature senescence of flag leaf severely affects wheat yield and quality. Chlorophyll (Chl) degradation is the most obvious symptom during leaf senescence and catalyzed by a series of enzymes. Pheophytin pheophorbide hydrolase (Pheophytinase, PPH) gene encodes a Chl degradation hydrolase.

**Results:**

In this study, the coding, genomic and promoter sequences of wheat *TaPPH-A* gene were cloned. The corresponding lengths were 1467 bp, 4479 bp and 3666 bp, respectively. Sequence structure analysis showed that *TaPPH-A* contained five exons and four introns. After the multiple sequences alignment of *TaPPH-A* genome from 36 accessions in a wheat diversity panel, four SNPs and one 2-bp InDel were observed, which formed two haplotypes, *TaPPH-7A-1* and *TaPPH-7A-2*. Based on the SNP at 1299 bp (A/G), a molecular marker *TaPPH-7A*-dCAPS was developed to distinguish allelic variation (A/G). Using the molecular markers, 13 SSR, and 116 SNP markers, a linkage map of chromosome 7A were integrated. *TaPPH-A* was mapped on the chromosome region flanked by *Xwmc9* (0.94 cM) and *AX-95634545* (1.04 cM) on 7A in a DH population. Association analysis between *TaPPH-7A* allelic variation and agronomic traits found that *TaPPH-7A* was associated with TGW in 11 of 12 environments and Chl content at grain-filling stage under drought stress using Population 1 consisted of 323 accessions. The accessions possessed *TaPPH-7A-1* (A) had higher TGW and Chl content than those possessed *TaPPH-7A-2* (G), thus *TaPPH-7A-1* (A) was a favorable allelic variation. By analyzing the frequency of favorable allelic variation *TaPPH-7A-1* (A) in Population 2 with 157 landraces and Population 3 with 348 modern cultivars, we found it increased from pre-1950 (0) to 1960s (54.5%), then maintained a relatively stable level about 56% from 1960s to 1990s.

**Conclusion:**

These results suggested the favorable allelic variation *TaPPH-7A-1* (A) should be valuable in enhancing grain yield by improving the source (chlorophyll content) and sink (the developing grain) simultaneously. Furthermore, the newly developed molecular marker *TaPPH-7A*-dCAPS could be integrated into a breeding kit of screening high TGW wheat for marker-assisted selection.

**Electronic supplementary material:**

The online version of this article (10.1186/s12870-019-1901-0) contains supplementary material, which is available to authorized users.

## Background

Wheat (*Triticum aestivum* L.) provides the staple food source for 30% human population in the world [1]. It is estimated that the global demand for wheat will increase by a further 40% before 2020, because of an increasing world population [2]. Therefore, breeding for high-yield varieties has still been a major objective in wheat breeding programs [3]. Thousand-grain weight (TGW) is an important yield-contributing trait. The significantly genetic improvement in wheat grain yield is partially attributed to increased TGW [4].

Delayed senescence, or stay-green, enables leaf to maintain a longer greenness after anthesis, and contributes to a longer grain-filling period [5, 6]. Gregersen, et al. [7] reported a positive correlation between delayed crop senescence and grain yield. A two-days delay in onset of senescence increased 11% carbon fixed in *Lolium temulentum* L [8]. Furthermore, the yield of wheat stay-green mutant *tasg1* was 9.5% higher than the wild type [9]. Thus it has been regarded as a desirable characteristic for the production of a number of crops including wheat. Chlorophyll (Chl) degradation is the main indicator of leaf senescence and catalyzed by a series of enzymes [10–14]. Pheophytin pheophorbide hydrolase (Pheophytinase, PPH) is a key enzyme in Chl degradation, specifically hydrolyzes pheophytin a to pheophorbide a [15, 16]. Mutagenesis or overexpression of *PPH* can lead to a stay-green or premature senescence phenotype in Arabidopsis and rice [15, 16]. In addition, overexpressing *LpPPH* also accelerated Chl degradation, and the expression level was positively related to leaf senescence [17]. Therefore, the expression of *PPH* gene affected Chl degradation, further affected yield and quality of crops.

Marker-assisted selection is considered a potential approach to accelerate the process of wheat breeding [18, 19]. Single nucleotide polymorphism (SNP) mainly refers to DNA sequence polymorphisms caused by single nucleotide changes and small insertions/deletions [20, 21]. Comparing with other marker types, such as RFLP, RAPD, AFLP, SSR and ISSR, SNP is a kind of more valuable marker due to its high abundance and relative stability, cost efficiency, and high-throughput scoring [22, 23]. Additionally, SNP has been also proved to be a kind of very effective molecular markers in high-density genetic map construction, fine mapping, genetic diversity, association analysis, functional marker development and marker-assisted selection breeding [24, 25]. Along with the development of high density genetic map of SNPs, association analysis has been proved to be an effective tool for identifying the relationship between polymorphic sites of target genes and important quantitative traits, and has been widely performed in many plant species, such as Arabidopsis [26, 27], rice [28, 29], maize [30–32], and wheat [33, 34].

**Fig. 1:**
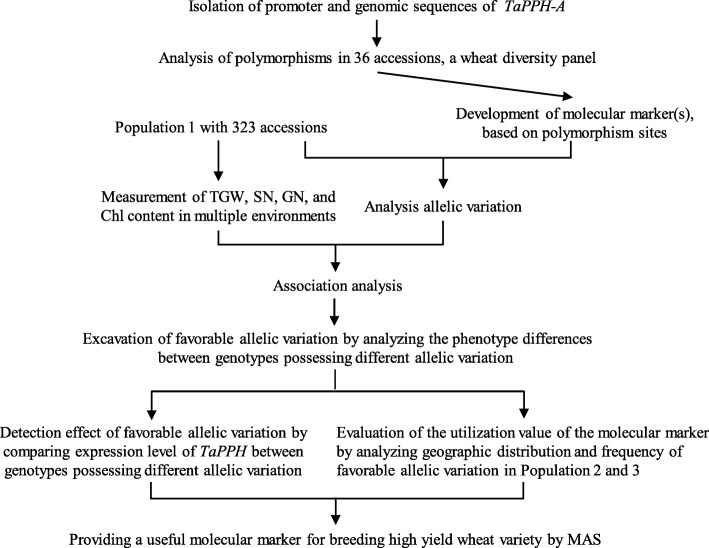
The scheme of the whole research work. TGW, thousand-grain weight; SN, spike number per plant; GN, grain number per spike; Chl, chlorophyll; MAS, marker-assisted selection

The *PPH* plays an important role during Chl degradation process. But the sequence, structure and polymorphism of *TaPPH* are still unclear in wheat. The objectives of this study were (i) to isolate the coding, genomic and promoter sequences of *TaPPH-A*, (ii) to identify polymorphism sites and develop molecular marker(s) from *TaPPH-A*, (iii) to identify favorable allelic variation, (iv) to evaluate the value of the newly molecular marker(s) by analyzing the geographic distribution and frequency of favorable allelic variation. The purpose is to provide a newly and effectively molecular marker for breeding high yield wheat variety by marker-assisted selection. The scheme of the whole research work is as follows (Fig. 1).

## Results

### Cloning and characterization of *TaPPH-A* gene

**Fig. 2:**
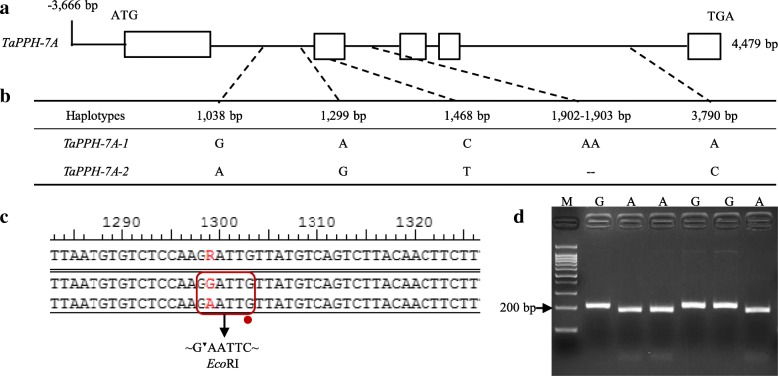
Single nucleotide polymorphisms and molecular marker of *TaPPH-7A.* (**a**) Schematic diagram of *TaPPH-7A* structure. (**b**) SNP and InDel in two haplotypes identified in *TaPPH-7A* among the wheat diversity panel. (**c**) The molecular marker *TaPPH-7A*-dCAPS, formed *Eco*RI restriction site by changing the base G to C. The *Eco*RI restriction site and a base G mismatched to C are marked in red rectangle and red point, respectively. (**d**) PCR products were digested by *Eco*RI. M is a 100 bp DNA Ladder

To obtain possible reference sequence of *TaPPH* gene, the nucleotide sequence of *OsNYC3* (Os06g0354700) was used as a query against Chinese Spring genome sequences database in the URGI (https://urgi.versailles.inra.fr/blast/blast.php). Three Chinese Spring wheat scaffold sequences (IWGSC_V3_chr7AL_scaffold_1626, IWGSC_V3_chr7BL_scaffold_507, IWGSC_V3_chr7DL_scaffold_2847) with high similarity (> 80%) were selected and identified as potential candidate sequence. The three scaffold sequences were downloaded, and used for nucleotide alignment through SeqMan. A genome-specific primer pair G-F/R and a promoter-specific primer pair P-F/R were designed, and used to clone the genomic and promoter sequences of *TaPPH-A* in Chang 4738. The corresponding lengths of the amplified fragments were 4125 bp and 4138 bp, respectively. Sequence analysis indicated that the genomic and promoter sequences of *TaPPH-A* were 4479 bp and 3666 bp in length, respectively. To analyze the gene structure of *TaPPH-A*, the coding sequence was cloned using a pair of primers C-F/R with the length of 1467 bp. Compared with the coding sequence, the genomic sequence of *TaPPH-A* consisted of five exons and four introns (Fig. 2a).

### Sequence polymorphism and molecular marker development for *TaPPH-A*

To detect polymorphisms in the genomic and promoter sequences of *TaPPH-A*, all accessions in wheat diversity panel were used to amplify the *TaPPH-A* gene, and the amplified fragments were sequenced. Multiple sequence alignment found that four SNPs (G/A, A/G, C/T, A/C) and a 2-bp InDel were observed in the genomic sequence, while no sequence variation was detected in the promoter sequence of *TaPPH-A* (Fig. 2a and b). From the start codon (ATG), four SNPs were located at 1038 bp, 1299 bp, 1468 bp, and 3790 bp, and the 2-bp InDel was identified from 1902 to 1903 bp. The four SNPs and the 2-bp InDel together formed two haplotypes, *TaPPH-7A-1* and *TaPPH-7A-2* (Fig. 2b).

Based on the SNP at 1299 bp (A/G), a molecular marker was developed and named *TaPPH-7A*-dCAPS. It contained a base G mismatched to C in the downstream primer *Eco*RI-R and a restriction enzyme *Eco*RI site (Fig. 2c). To characterize the observed *TaPPH-A* allelic variation in large wheat populations, the following three experiments were performed: firstly, the genome-specific primer pair, G-F/R, was used to amplify genomic sequence of *TaPPH-A*, and a 4125 bp fragment was obtained. Secondly, 0.2 μL PCR product of 4125 bp was used as the template to amplify a 202 bp fragment by the molecular marker primer pair, *Eco*RI-F/R. Thirdly, the PCR product of 202 bp possessed allelic variation *TaPPH-7A-1* (A) formed a *Eco*RI site GAATTC (Fig. 2c), and was digested into 174 bp and 28 bp by restriction enzyme *Eco*RI (Fig. 2d). Whereas the corresponding PCR product possessed allelic variation *TaPPH-7A-2* (G) was GGATTC (Fig. 2c), and corresponding product remained 202 bp (Fig. 2d).

### Genetic mapping of *TaPPH*

**Fig. 3:**
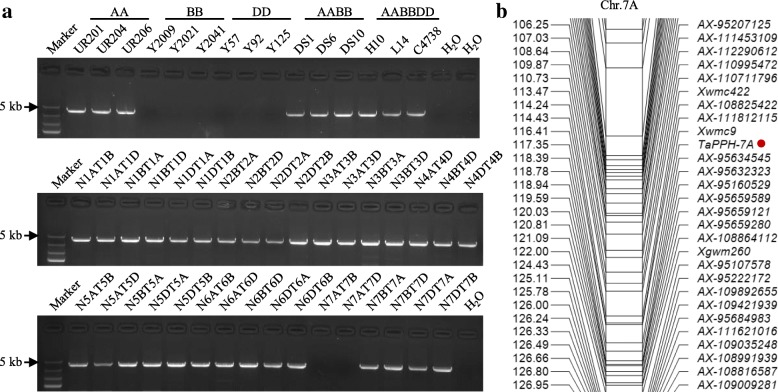
Chromosome location and genetic mapping of *TaPPH.* (**a**) Location of *TaPPH* on chromosome 7A using 15 wheat species of different ploidy and 33 nulli-tetrasomic lines of Chinese Spring. H10, Hanxuan 10; L14, Lumai 14; C4738, Chang 4738. (**b**) Linkage map of *TaPPH* on wheat chromosome 7A flanked by SSR marker *Xwmc9* and SNP marker *AX-95634545* in the DH population. The target location is marked by red point

Fifteen wheat species of different ploidy and a set of Chinese Spring nulli-tetrasomic lines were used to assign chromosomal location to *TaPPH-A*. Target fragment was only detected in accessions possessing chromosomes 7A, including three AA genome accessions, three AABB genome accessions, three AABBDD genome accessions, and all nulli-tetrasomic lines besides N7AT7B and N7AT7D of Chinese Spring (Fig. 3a). This indicated that *TaPPH* was located on chromosome 7A. To further map its position, the molecular marker, 13 SSR and 116 SNP markers were integrated into a linkage map of chromosome 7A. Using the DH population derived from the cross of Hanxuan 10 × Lumai 14, *TaPPH* was mapped on the chromosome region flanked by *Xwmc9* (0.94 cM) and *AX-95634545* (1.04 cM) on 7A (Fig. 3b). Therefore, it was named as *TaPPH-7A*.

### Association of *TaPPH-7A* with yield-related traits

**Fig. 4:**
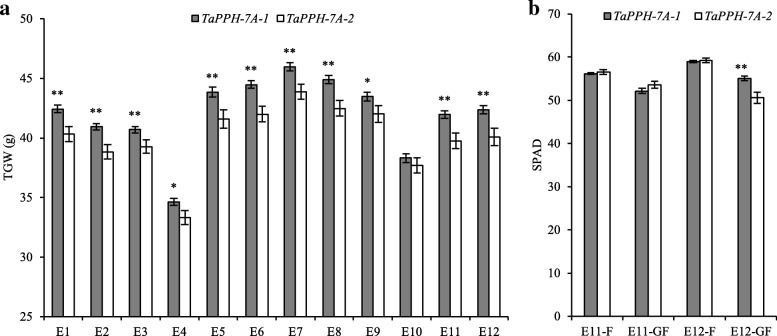
Agronomic traits comparisons between genotypes possessing different *TaPPH-7A* allelic variation. Traits are TGW (**a**) and Chl content (**b**). TGW, thousand-grain weight; E1 to E12 indicate the environments of 2015-SY-WW, 2015-SY-DS, 2015-SY-WW + HS, 2015-SY-DS + HS, 2016-CP-WW, 2016-CP-DS, 2016-SY-WW, 2016-SY-DS, 2016-SY-WW + HS, 2016-SY-DS + HS, 2017-SY-WW and 2017-SY-DS, respectively. F, Flowering stage; GF, Grain filling stage. SY, Shunyi; CP, Changping; WW, Well-watered; DS, Drought stress; HS, Heat stress. ^*^P < 0.05, ^**^P < 0.01, respectively. Error bars denote SE

To detect association of *TaPPH-7A* allelic variation with yield-related traits, the Population 1 consisted of 323 wheat accessions was used. In Population 1, *TaPPH-7A-1* (A) was a major allelic variation, accounting for 69.3% of the total accession number (Additional file 1: Figure S1). *TaPPH-7A* had no significant association with GN in all 12 environments, and no significant association with SN in seven environments (E1, E2, E3, E5, E6, E7, and E10) (Table 1). However, significant association between *TaPPH-7A* and TGW was observed in 11 of 12 environments, except for E10 (Table 1). Genotypes possessing allelic variation *TaPPH-7A-1* (A) had significantly higher TGW than those possessing allelic variation *TaPPH-7A-2* (G) (Fig. 4a). Thus, *TaPPH-7A-1* (A) could be a favorable allelic variation for improving TGW.

**Table 1:** Association analysis of *TaPPH-7A* allelic variation and yield-related traits in 12 environments

Environment	SN (*P*-value)	GN (*P*-value)	TGW (*P*-value)
E1	0.11	0.65	8.65E-04 ^**^
E2	0.91	0.15	3.56E-04 ^**^
E3	0.84	0.39	0.01 ^*^
E4	4.56E-04 ^**^	0.13	0.02 ^*^
E5	0.83	0.36	3.10E-03 ^**^
E6	0.69	0.92	7.60E-05 ^**^
E7	0.15	0.80	1.90E-03 ^**^
E8	2.50E-03 ^**^	0.13	2.32E-04 ^**^
E9	6.14E-04 ^**^	0.86	0.04 ^*^
E10	0.26	0.07	0.41
E11	0.02 ^*^	0.36	4.97E-04 ^**^
E12	3.39E-04 ^**^	0.33	8.43E-04 ^**^

SN, spike number per plant; GN, grain number per spike; TGW, thousand-grain weight; E1 to E12, the environments of 2015-SY-WW, 2015-SY-DS, 2015-SY-WW + HS, 2015-SY-DS + HS, 2016-CP-WW, 2016-CP-DS, 2016-SY-WW, 2016-SY-DS, 2016-SY-WW + HS, 2016-SY-DS + HS, 2017-SY-WW and 2017-SY-DS, respectively. SY, Shunyi; CP, Changping; WW, Well-watered; DS, Drought stress; HS, Heat stress. ^*^P < 0.05 and ^**^P < 0.01, respectively

### Association of *TaPPH-7A* with Chl content

In Population 1, we also found that *TaPPH-7A* had a significant association with Chl content at grain-filling stage under drought stress (Table 2). Genotypes possessing allelic variation *TaPPH-7A-1* (A) had significantly higher Chl content than those of possessing allelic variation *TaPPH-7A-2* (G) (Fig. 4b). So *TaPPH-7A-1* (A) might be a superior allelic variation to delay Chl degradation at grain-filling stage under drought stress.

**Table 2:** Association analysis of *TaPPH-7A* allelic variation and Chl contents in two environments

Environment	Chl (*P*-value)
E11-F	0.30
E11-GF	0.22
E12-F	0.49
E12-GF	1.46E-04 ^**^

Chl, Chlorophyll content; E11 and E12, the environment of 2017-SY-WW and 2017-SY-DS; F, Flowering stage; GF, Grain filling stage. SY, Shunyi; WW, Well-watered; DS, Drought stress. ^*^P < 0.05 and ^**^P < 0.01, respectively

### Quantitative real-time PCR analysis of *TaPPH-7A* expression

**Fig. 5:**
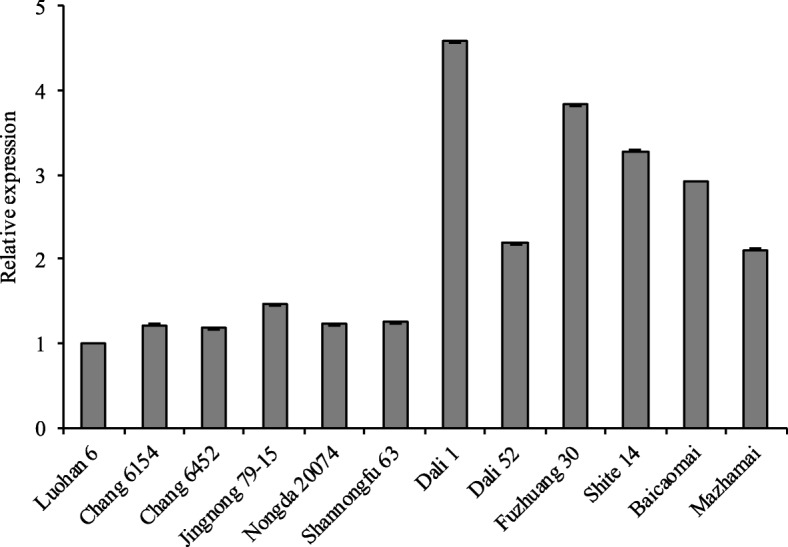
Relative expression levels of two *TaPPH-7A* allelic variation in leaves at grain filling stage. The first six genotypes, Luohan 6, Chang 6154, Chang 6452, Jingnong 79–15, Nongda 20,074, and Shannongfu 63 possess the allelic variation *TaPPH-7A-1* (A). The second six genotypes, Dali 1, Dali 52, Fuzhuang 30, Shite 14, Baicaomai, and Mazhamai possess the allelic variation *TaPPH-7A-2* (G). Error bars denote SE

To detect effect of favorable allelic variation on TGW and Chl content, six accessions each possessing *TaPPH-7A-1* (A) and *TaPPH-7A-2* (G) were randomly selected from Population 1, and used to test the expression level of *TaPPH-7A* (Additional file 3: Table S1). The melting curves of qRT-PCR for genes *TaPPH-7A* and *TaActin* were both single peaks, indicating primer specificity of the amplification (Additional file 2: Figure S2). qRT-PCR revealed that genotypes with *TaPPH-7A-1* (A) had lower relative expression level than those with *TaPPH-7A-2* (G) (Fig. 5). This finding suggested that lower relative expression level of *TaPPH-7A-1* might help to maintain a higher Chl content, thus synthesizing more photosynthates, furthermore improving TGW.

### Geographic distribution of *TaPPH-7A* allelic variation in Chinese wheat production zones

**Fig. 6:**
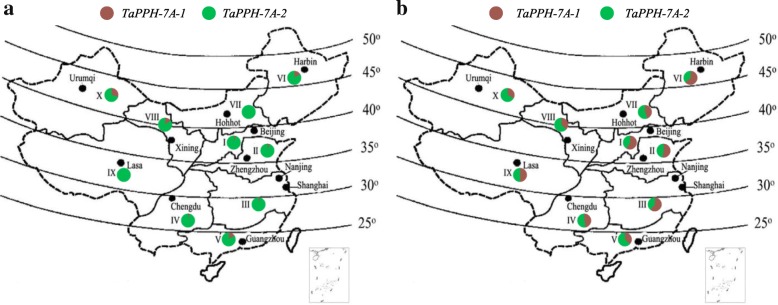
Geographic distribution of *TaPPH-7A* allelic variation in Chinese landraces and modern cultivars of wheat. (**a**) Geographic distribution of *TaPPH-7A* allelic variation in 157 Chinese landraces. (**b**) Geographic distribution of *TaPPH-7A* allelic variation in 348 modern cultivars. I, Northern Winter Wheat Zone; II, Yellow and Huai River Valleys Facultative Wheat Zone; III, Middle and Lower Yangtze Valleys Autumn-sown Spring Wheat Zone; IV, Southwestern Autumn-sown Spring Wheat Zone; V, Southern Autumn-sown Spring Wheat Zone; VI, Northeastern Spring Wheat Zone; VII, Northern Spring Wheat Zone; VIII, Northwestern Spring Wheat Zone; IX, Qinghai-Tibetan Plateau Spring-Winter Wheat Zone; X, Xinjiang Winter-Spring Wheat Zone. The maps were generated using Mapinfo Professional software

In China, ten wheat Zones were divided according to wheat types, growing seasons, varietal response to photoperiod, temperature, moisture, biotic and abiotic stresses [35]. In this study, all accessions of Population 2 with 157 landraces and Population 3 with 348 modern cultivars were collected from the ten wheat Zones (Additional file 4: Table S2). The geographic distribution of *TaPPH-7A* allelic variation was evaluated using Population 2 and Population 3. In Population 2, only 12 accessions (7.6%) possessed favorable allelic variation *TaPPH-7A-1* (A), and the frequency was much lower than that of *TaPPH-7A-2* (G) (Additional file 1: Figure S1). The largest frequency of *TaPPH-7A-1* (A) was 30.0% in Zone X, followed by 16.7% in Zones V and VI. In Population 3, favorable allelic variation *TaPPH-7A-1* (A) was identified in 183 accessions (52.6%) (Additional file 1: Figure S1), and the frequencies in all ten wheat Zones were higher than those in Population 2. In Zones I, II, III and VI, which accounted for about 75% China wheat area, the frequencies of *TaPPH-7A-1* (A) were higher than that of *TaPPH-7A-2* (G), but in other wheat growing areas, such as Zones V, VIII and X, the frequencies of *TaPPH-7A-1* (A) were obviously lower than *TaPPH-7A-2* (G) (Additional file 4: Table S2, Fig. 6a and b). Thus, favorable allelic variation *TaPPH-7A-1* (A) was strongly selected in the major wheat production regions, and weakly selected in other wheat production regions.

### The frequency of *TaPPH-7A* allelic variation in wheat breeding history

**Fig. 7:**
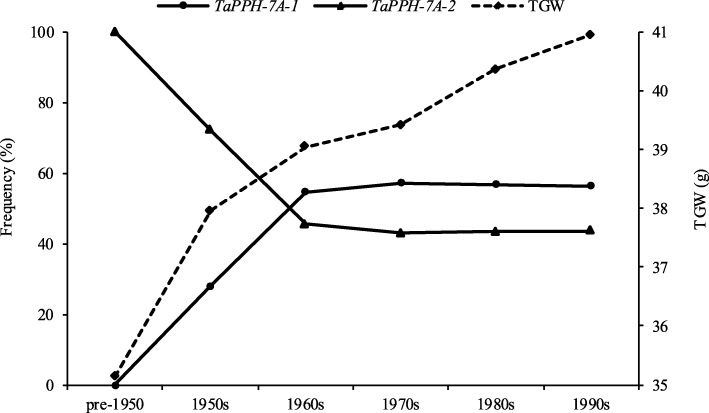
The frequencies of *TaPPH-7A* allelic variation and TGW in wheat accessions released in different era

To evaluate the frequency of favorable allelic variation *TaPPH-7A-1* (A), 334 accessions known released dates from Population 3 were used and divided into six groups (pre-1950, 1950s, 1960s, 1970s, 1980s and 1990s). All eight accessions, which released before 1950 (pre-1950), possessed the allelic variation *TaPPH-7A-2* (G). The frequency of *TaPPH-7A-1* (A) increased from pre-1950 (0) to 1960s (54.5%), then maintained a relatively stable level about 56% from 1960s to 1990s (Fig. 7). The TGW exhibited a trend of continuous increase from pre-1950 to 1990s (Fig. 7). These strongly indicated that favorable allelic variation *TaPPH-7A-1* (A) should be valuable, and could be selected to improve TGW.

## Discussion

### Sequence polymorphism of *TaPPH-7A*

During the evolution, domestication and breeding in wheat, the genetic information had undergone two doublings and mutations under natural conditions, which eventually resulted in a rich genetic diversity. Somers, et al. [36] reported the level of sequence polymorphism was 1 SNP every 540 bp of EST sequence using a bioinformatics strategy based on a large wheat EST database from 12 cultivars. Ravel, et al. [37] identified that the SNP frequency was 1 SNP/334 bp in the genomic sequence including coding and non-coding region, and 1 SNP/267 bp in coding region in wheat. In this study, polymorphisms of *TaPPH-7A* gene sequences from the wheat diversity panel (36 accessions) was detected by direct sequencing. The SNPs occurred in both exons and introns region of *TaPPH-7A* (Fig. 2). This study allowed us to estimate an average of 1 SNP for every 1120 bp in the genomic sequence including coding and non-coding region, and 1 SNP every 1467 bp in coding region, 1 SNP every 1004 bp in non-coding region, but no polymorphism detected in the promoter region. As expected, the coding region had lower SNP frequency than the non-coding region, and this result is consistent with earlier studies [38]. However, the SNP frequency of the whole genomic sequence was much lower than those in the previous studies [36, 37]. This result suggested that *TaPPH-7A* is a relatively conservative gene in evolution.

In this study, only one synonymous mutation SNP (C/T) was identified in the exon region at 1468 bp of *TaPPH-7A*. However, the *TaPPH-7A* associated with TGW and Chl content, and the relative expression levels of *TaPPH-7A* were lower in wheat genotypes with *TaPPH-7A-1* (A) than those with *TaPPH-7A-2* (G) (Fig. 5), so the reason for the phenotypic and gene expression variation may be alternative splicing. Many previous studies had showed that variations in the introns can significantly affect gene expression or phenotypic variation by alternative splicing of pre-mRNA [39–42]. Thus, variations in introns are important and worthy of future investigation.

### *TaPPH-7A* is a novel locus related to TGW and Chl content

Using QTL mapping, it is possible to dissect loci controlling genetic variation and characterize these loci based on map position, phenotypic effects, gene actions, pleiotropic effects, and epistatic interactions with other QTL in a segregating population [43]. Several QTL for Chl content and TGW were detected on wheat chromosome 7A in various mapping populations. Using the DH population derived from the cross of Hanxuan 10 × Lumai 14, Shi, et al. [44] found two QTL for Chl content at flowering and grain-filling stage on chromosome 7A with 7.32 and 8.36% of phenotypic variation, respectively, and Yang, et al. [45] detected a QTL for Chl content in the interval *Xwmc488*-*P2071–180* on chromosome 7A under water deficient. Bhusal, et al. [46] also reported a QTL for Chl *a* content on chromosome 7A close to the marker *Xwmc388* under heat stress. Ilyas, et al. [47] mapped a QTL for total Chl content on 7A flanked by *Xbarc49* and *Xgdm14*. As for TGW, a few QTL have been reported on chromosome 7AL in wheat, too. For example, Wang, et al. [48] reported a QTL for TGW close to the marker *Xcfa2257* in a natural population, which explained up to 21.99% of the total phenotypic variation. Groos, et al. [49] also found a QTL for TGW in the vicinity of *Xgwm282*, explaining phenotypic variation ranging from 5.2 to 10.3% in six environments. In the present study, *TaPPH* was located on chromosome 7A between markers *Xwmc9* and *AX-95634545* within an interval of 1.98 cM by QTL mapping (Fig. 3b). Based on the common wheat consensus maps [50, 51], *TaPPH-7A* is considered to be near to the centromere, but is far from markers *Xwmc488* and *P2071–180* [45], *Xwmc388* [46], *Xbarc49* and *Xgdm14* [47], *Xcfa2257* [48], and *Xgwm282* [49]. Thus, *TaPPH-7A* was likely a novel locus related to Chl content and TGW on chromosome 7A.

### *TaPPH-7A*-dCAPS is a stably and effectively molecular marker for assisted selection breeding

Functional molecular marker was developed from polymorphic site within gene causally affecting phenotypic trait variation [52]. Converting SNPs to CAPS or dCAPS markers enable SNPs to be more conveniently applied in selecting preferred alleles in marker-assisted breeding [18]. In addition, the assay procedure of CAPS or dCAPS marker was simple. However, it is difficult to develop CAPS or dCAPS markers from identified wheat genes because of the large allohexaploid genome [53, 54]. In order to solve this problem, we firstly designed genome-specific primer to distinguish three ortholog genome sequences, then identified SNPs by comparing the genomic sequences of target gene from different wheat genotypes, finally developed molecular markers based on SNP. Using the method described above, a molecular marker *TaPPH-7A*-dCAPS was developed based on the SNP at 1299 bp in this study (Fig. 2c). Two allelic variation, *TaPPH-7A-1* (A) and *TaPPH-7A-2* (G), were detected by scanning the Population 1 using the developed molecular marker (Additional file 5: Table S3). We made further efforts to found that favorable allelic variation *TaPPH-7A-1* (A) was associated with high TGW in 11 of 12 environments and Chl content at grain-filling stage under drought stress using Population 1 (Fig. 4). And we also found that genotypes with favorable allelic variation *TaPPH-7A-1* (A) had lower relative expression level of *TaPPH-7A* than those with *TaPPH-7A-2* (G) (Fig. 5). Therefore, it is deduced that the favorable allelic variation, *TaPPH-7A-1* (A), could simultaneously improve the source (chlorophyll content) and sink (the developing grain) after flowering, and eventually contribute to wheat grain weight. Thus, the newly developed molecular marker, *TaPPH-7A*-dCAPS, is a stable and effectively molecular marker for grain yield, and can be used to marker-assisted selection breeding in wheat.

### Favorable allelic variation *TaPPH-7A-1* (a) was selected in wheat breeding history

Wheat is one of the most important staple food crops in China. With the development of economy, the breeding objective is constantly changing. Before 1960s, the main breeding objective was to increase TGW and further improve yield. From 1970s to 1990s, the breeding objective was changed to improve agronomic traits such as plant height, quality, spike number and grain weight per spike [55]. The change of Chinese wheat breeding objective from 1950 to 1960s resulted in the positive selection of favorable allelic variation *TaPPH-7A-1* (A) and the rapid increase in TGW before 1970 (Fig. 7). From 1970s to 1990s, the frequency of favorable allelic variation *TaPPH-7A-1* (A) maintained a relatively stable level about 56%, but TGW continuously increased (Fig. 7), the possible reason may be that other genes contributing to TGW were selected, such as *TaSnRK2.3-1A* (*Hap-1A-1*) and *TaSnRK2.3-1B* (*Hap-1B-1*) [56], *TaSPL21-6D-Hap*II&III [57], and *TaSAP7-B* (C) [58]. Moreover, the frequency of favorable allelic variation *TaPPH-7A-1* (A) was increased from landraces (Population 2) to modern cultivars (Population 3) in Chinese ten wheat Zones. However, in Population 3, the maximum frequency of *TaPPH-7A-1* (A) was only 63.6% in Zone VI (Additional file 4: Table S2, Fig. 6a and b). Thus, there is a large potential for increasing TGW by selecting favourable allelic variation *TaPPH-7A-1* (A) in high-yield wheat breeding.

## Conclusions

The *TaPPH-7A* gene was cloned. Four SNPs and one 2-bp InDel were observed. A molecular marker *TaPPH-7A*-dCAPS was developed based on a SNP at 1299 bp (A/G). Favourable allelic variation *TaPPH-7A-1* (A) was found to be associated with high TGW and Chl content, but not with SN and GN in Population 1. The frequency of favourable allelic variation *TaPPH-7A-1* (A) was maintained a relatively stable level of about 56% from 1960s to 1990s in Population 3, thus the favourable allelic variation should be valuable, and could be selected to increase grain yield by improving the source (chlorophyll content) and sink (the developing grain) simultaneously. The newly developed molecular marker *TaPPH-7A*-dCAPS could be integrated into a breeding kit of screening high TGW wheat for marker-assisted selection.

## Methods

### Plant materials

Chang 4738, a wheat variety with a high TGW and a slowly chlorophyll degradation rate after anthesis, was used to clone *TaPPH-7A* gene. A wheat diversity panel (36 accessions, Additional file 6: Table S4) was chosen to detect polymorphisms in *TaPPH-7A* genomic sequence. Fifteen wheat species of different ploidy and a set of nulli-tetrasomic lines of Chinese Spring were used for chromosome location of *TaPPH*. A DH population [59], derived from a cross of Hanxuan 10 × Lumai 14, consisting of 150 lines, was used for linkage mapping of *TaPPH*.

Three wheat germplasm populations were used as plant materials to analyze the allelic variation in *TaPPH-7A*. Population 1 consisted of 323 accessions (Additional file 3: Table S1), including 275 modern cultivars, 36 advanced lines, and 12 landraces, was used for association analysis of the target gene allelic variation and phenotypic traits. Population 2 (157 landraces) and Population 3 (348 modern cultivars) [35] (provided by Dr. Xueyong Zhang, at Chinese Academy of Agricultural Sciences) were used to analyze the frequency of favorable allelic variation in different wheat production zones and wheat breeding history, and to evaluate the utilization value of the newly developed molecular markers.

All wheat accessions were legally obtained from Chinese Crop Germplasm Resources Information System (http://www.cgris.net/zhongzhidinggou/index.php).

### Field management and phenotypic assessment

Population 1 was planted at Experiment Stations at Changping (116°13′E; 40°13′N) and Shunyi (116°56′E; 40°23′N) in Beijing over 3 growing seasons, i.e. in 2015 at Shunyi, 2016 at Changping and Shunyi, and 2017 at Shunyi. The experiment field was divided into two plots with different water regimes: rain-fed (drought stressed, DS) and well-watered (WW). The DS plots were not irrigated during the whole growing season but had rainfalls of 173 mm, 143 mm, and 116 mm, respectively. The WW plots were irrigated with 750 m^3^/ha (75 mm): before winter, at booting, flowering and grain filling when the amounts of rainfall were insufficient during each corresponding period. In addition, a heat stress experiment (HS) was conducted by adding polythene covers over the plots at Shunyi in 2015 and 2016. The other field managements, such as fertilization, disease and pest control were the same as local production conditions. Phenotypic assessment was performed under 12 environments (E1 to E12). E1 to E12 indicated the environments at Shunyi in 2015 under WW, DS, WW + HS and DS + HS, Changping in 2016 under WW and DS, Shunyi in 2016 under WW, DS, WW + HS and DS + HS, Shunyi in 2017 under WW and DS, respectively.

Yield-related traits, including spike number per plant (SN), grain number per spike (GN) and TGW, were measured under 12 environments. Chl content (SPAD value) was tested with a handheld portable chlorophyll meter (SPAD-502, Konica-Minolta, Tokyo) at flowering and grain filling stages in two environments (E11 and E12) (Additional file 7: Table S4). The SN, GN, TGW and Chl content were measured using five plants, separately.

Population 3 was grown at Luoyang (112°45′E; 36°61′N) in Henan Province during the 2001–2002 and 2004–2005, and Shunyi (116°56′E; 40°23′N) in Beijing during 2009–2010 cropping seasons [60]. TGW was measured in all the three environments.

### DNA and RNA extraction and *TaPPH-A* gene cloning

Seedlings of Chang 4738 were used as experimental materials. Genomic DNA was extracted by the CTAB method [61]. Total RNA was extracted using an RNAprep Pure Plant Kit (Tiangen, Beijing) following the manufacturer’s instructions. First-strand cDNA was synthesized with a FastQuant RT Kit (with gDNase) (Tiangen, Beijing).

The nucleotide sequence of the rice *NYC3* (Os06g0354700) gene published on China Rice Data Center (http://www.ricedata.cn/gene/index.htm) was used for a blast search against the reference sequence of Chinese Spring from URGI Blast website (https://urgi.versailles.inra.fr/blast/blast.php). All Chinese Spring wheat scaffold sequences with high similarity to *OsNYC3* sequence were downloaded. According to the scaffold sequences, three pairs of 7A-specific primers were designed using the software Primer Premier 5.0. All primers were synthesized by Beijing Huada Genomics, and listed in Table 3. The first pair of 7A-specific primers, C-F/R, was used to amplify coding sequence of *TaPPH-A*. The second pair of 7A-specific primers, G-F/R, was used to amplify genomic sequence of *TaPPH-A*. The third pair of 7A-specific primers, P-F/R, was used to amplify the promoter sequence of *TaPPH-A*.

**Table 3:** Primer sequences used in this study

Primer name	Primer sequence (5′-3′)	Function
C-F	ATGGAAGTGGTTTCTTCCAGTCAC	Cloning coding sequence
C-R	TCATCTGGATACTACCCGTATGC	
G-F	GGCACCAAGAATAGCAAGGC	Cloning *TaPPH-A* genomic specific sequence
G-R	TCATCTGGACACTACCTGTATGTTGGAGG	
P-F	TGGTTCGCAGGGATGACTGTAAC	Cloning *TaPPH-A* promoter specific sequence
P-R	CCATCGTCCACACCTTGTAATCA	
RT-F	TATTGGGGTATCAGAGTCAAGCAG	Expression analysis
RT-R	AATCATCTGGATACTACCCGTATGC	
RT-Actin-F	CTCCCTCACAACAACAACCGC	Endogenous control
RT-Actin-R	TACCAGGAACTTCCATACCAAC	
*Eco*RI-F	AAGTCTTCGTTGGTGCTCAC	Marker *TaPPH-7A*-dCAPS developed for SNP-1299 (A/G)
*Eco*RI-R	AAGAAGTTGTAAGACTGACATAAGAAT	

The cDNA and genomic DNA of Chang 4738 were used as templates. PCR amplification was performed in 20 μL volume including 1 μL 50 ng/μL cDNA or genomic DNA, 0.2 μM of each primer, 0.2 mM dNTPs, 4 μL 5 × *TransStart****®*** *FastPfu* Buffer, and 0.4 μL (2.5 U/μL) *TransStart****®*** *FastPfu* DNA Polymerase (TransGen Biotech, Beijing). The amplification program consisted of an initial denaturation at 95 °C for 5 min, followed by 35 cycles of denaturation at 95 °C for 30 s, annealing at 58 °C for 45 s, and extension at 72 °C for 1–2 min, with a final extension at 72 °C for 15 min. The PCR products were separated by electrophoresis in 1.2% agarose gel. The target bands were purified using Gel Extraction Kit (BIOMIGA, China). The purified PCR product was cloned into *pEASY***®**-Blunt cloning vector (TransGen Biotech, Beijing), then was transformed to 33 μL *Trans*1-T1 Phage Resistant Chemically Competent Cells (TransGen Biotech, Beijing) by heat shock. Six positive clones of each wheat genotype were sequenced. The sequence alignment was performed using SeqMan (DNASTAR Lasergene 7.1.0). The gene structure of *TaPPH-A* was determined using MegAlign (DNASTAR Lasergene 7.1.0) through alignment the coding and genomic sequences.

### Molecular marker development

The sequences of *TaPPH-7A* cloned from the wheat diversity panel were aligned by SeqMan for screening polymorphism. Molecular marker(s) were developed based on polymorphism sites. A dCAPS marker was designed using dCAPS Finder 2.0 (http://helix.wustl.edu/dcaps/dcaps.html). The primer pair of dCAPS marker was *Eco*RI-F/R. Genotyping was performed by two rounds of PCR and one enzyme digestion following the procedure described by Miao, et al. [56].

### Population structure and association analysis

Population 1 was performed on Wheat 660 K SNP Array, which consisted of 630,517 SNPs [62]. By removing nucleotide variations with missing rates ≥0.2 and minor allele frequency (< 0.05), 395,681 SNPs were eventually used to detect the structure of Population 1 by software STRUCTURE 2.3.4 [59]. A general linear model was performed in TASSEL 2.1 for analyzing significant associations between the target gene allelic variation and and phenotypic traits. Associations were considered significant at *P* < 0.05. Statistical analysis was conducted by SPSS 19.0 software (SPSS Corp., Chicago, IL, USA).

### Analysis of gene expression with quantitative real-time PCR

A primer pair RT-F/R (Table 3) was designed to use for analyzing the expression of *TaPPH-7A* in different wheat genotypes. Quantitative real-time PCR (qRT-PCR) was performed in triplicate with Roche LightCycler® 96 using the SYBR® *Premix Ex Taq*™ (Tli RNaseH Plus) (Takara, Japan). The qRT-PCR reaction system with specific primer contains 10 μL 2 × SYBR Premix Ex Taq™, 0.4 μL 50 × Rox Reference Dye II, 0.4 μL (5 μM) of each primer (Table 3), 1 μL cDNA template, and 7.8 μL ddH_2_O. The reaction procedure was as follows: denaturation at 95 °C for 2 min; followed by 45 cycles at 95 °C for 20 s, 60 °C for 20 s, and 72 °C for 20 s. *TaActin* was used as the endogenous control of normalizing expression levels of different samples. Gene relative expression levels were calculated using the 2^-△△*C*T^ method [63]. The statistical analysis of ΔΔ*C*_T_ according to the method described by Zhang et al. [35].

## Additional files

Additional file 1: **Figure S1.** Frequencies of *TaPPH-7A* allelic variation in Populations 1–3. (DOCX 16 kb)

Additional file 2: **Figure S2.** The melting curves of qRT-PCR for genes *TaPPH-7A* and *TaActin*. The blue and red lines indicate *TaPPH-7A* and *TaActin*, respectively. (DOCX 60 kb)

Additional file 3: **Table S1.** The information of 12 wheat varieties for qRT-PCR analysis (DOCX 14 kb)

Additional file 4: **Table S2.** The frequencies (%) of *TaPPH-7A* allelic variation in Populations 2 and 3 from ten Chinese wheat production zones (DOCX 16 kb)

Additional file 5: **Table S3.** The information of Population 1 and their genotypes of *TaPPH-7A (DOCX 51 kb)*

Additional file 6: **Table S4.** The information of the wheat diversity panel and their genotypes of *TaPPH-7A (DOCX 28 kb)*

Additional file 7 **Table S5.** Statistical analysis on traits of Population 1 (DOCX 28 kb)

## Data Availability

All data generated or analysed during this study are included in this published article [and its Additional files].

## Abbreviations

Chl: Chlorophyll
dCAPS: Derived cleaved amplified polymorphic sequence
DH: Doubled haploid
DS: Drought stress
GN: Grain number per spike
PPH: Pheophytin pheophorbide hydrolase
QTL: Quantitative trait loci
SN: Spike number per plant
SNP: Single nucleotide polymorphism
TGW: Thousand-grain weight
WW: Well-watered
