# Supplementary material for: Identification of TaPPH-7A haplotypes and development of a molecular marker associated with important agronomic traits in common wheat
Source: BMC Plant Biol. 2019 Jul 8;19:296. doi: 10.1186/s12870-019-1901-0 (PMC6615193; doi:10.1186/s12870-019-1901-0)
Supplement: Supplementary file 1 — Figure S1. Frequencies of TaPPH-7A allelic variation in Populations 1–3. (DOCX 16 kb) [file 12870_2019_1901_MOESM1_ESM.docx]

**Additional file 1: Figure S1.** Frequencies of *TaPPH-7A* allelic variation in Populations 1-3.
