# Supplementary material for: Identification of TaPPH-7A haplotypes and development of a molecular marker associated with important agronomic traits in common wheat
Source: BMC Plant Biol. 2019 Jul 8;19:296. doi: 10.1186/s12870-019-1901-0 (PMC6615193; doi:10.1186/s12870-019-1901-0)
Supplement: Supplementary file 2 — Figure S2. The melting curves of qRT-PCR for genes TaPPH-7A and TaActin. The blue and red lines indicate TaPPH-7A and TaActin, respectively. (DOCX 60 kb) [file 12870_2019_1901_MOESM2_ESM.docx]

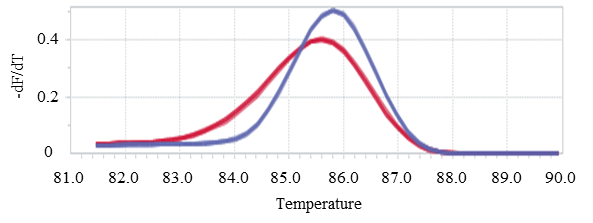


**Additional file 2: Figure S2.** The melting curves of qRT-PCR for genes *TaPPH-7A* and *TaActin*. The blue and red lines indicate *TaPPH-7A* and *TaActin*, respectively.
