# Supplementary material for: Identification of TaPPH-7A haplotypes and development of a molecular marker associated with important agronomic traits in common wheat
Source: BMC Plant Biol. 2019 Jul 8;19:296. doi: 10.1186/s12870-019-1901-0 (PMC6615193; doi:10.1186/s12870-019-1901-0)
Supplement: Supplementary file 3 — Table S1. The information of 12 wheat varieties for qRT-PCR analysis (DOCX 14 kb) [file 12870_2019_1901_MOESM3_ESM.docx]

**Additional file 3: Table S1.** The information of 12 wheat varieties for qRT-PCR analysis

| Number | Accession | Allele | Number | Accession | Allele |
| --- | --- | --- | --- | --- | --- |
| 34 | Luohan 6 | A | 5 | Dali 1 | G |
| 129 | Chang 6154 | A | 6 | Dali 52 | G |
| 131 | Chang 6452 | A | 11 | Fuzhuang 30 | G |
| 202 | Jingnong 79-15 | A | 75 | Shite 14 | G |
| 247 | Nongda 20074 | A | 111 | Baicaomai | G |
| 258 | Shannongfu 63 | A | 240 | Mazhamai | G |
