# Supplementary material for: Identification of TaPPH-7A haplotypes and development of a molecular marker associated with important agronomic traits in common wheat
Source: BMC Plant Biol. 2019 Jul 8;19:296. doi: 10.1186/s12870-019-1901-0 (PMC6615193; doi:10.1186/s12870-019-1901-0)
Supplement: Supplementary file 4 — Table S2. The frequencies (%) of TaPPH-7A allelic variation in Populations 2 and 3 from ten Chinese wheat production zones (DOCX 16 kb) [file 12870_2019_1901_MOESM4_ESM.docx]

**Additional file 4: Table S2.** The frequencies (%) of *TaPPH-7A* allelic variation in Populations 2 and 3 from ten Chinese wheat production zones

| Zone | Population 2 | | | Population 3 | | |
| --- | --- | --- | --- | --- | --- | --- |
|  | *TaPPH-7A-1* | *TaPPH-7A-2* | Total number | *TaPPH-7A-1* | *TaPPH-7A-2* | Total number |
| I | 1 (5.3) | 18 (94.7) | 19 | 33 (58.9) | 23 (41.1) | 56 |
| II | 2 (5.7) | 33 (94.3) | 35 | 62 (53.0) | 55 (47.0) | 117 |
| III | 1 (4.3) | 22 (95.7) | 23 | 31 (60.8) | 20 (39.2) | 51 |
| IV | 1 (4.5) | 21 (95.5) | 22 | 19 (47.5) | 21 (52.5) | 40 |
| V | 1 (16.7) | 5 (83.3) | 6 | 3 (37.5) | 5 (62.5) | 8 |
| VI | 1 (16.7) | 5 (83.3) | 6 | 14 (63.6) | 8 (36.4) | 22 |
| VII | 0 (0) | 9 (100.0) | 9 | 4 (44.4) | 5 (55.6) | 9 |
| VIII | 2 (14.3) | 12 (85.7) | 14 | 12 (37.5) | 20 (62.5) | 32 |
| IX | 0 (0) | 13 (100.0) | 13 | 2 (50.0) | 2 (50.0) | 4 |
| X | 3 (30.0) | 7 (70.0) | 10 | 3 (33.3) | 6 (66.7) | 9 |
| **Total** | **12 (7.6)** | **145 (92.4)** | **157** | **183 (52.6)** | **165 (47.4)** | **348** |

I, Northern Winter Wheat Zone; II, Yellow and Huai River Valleys Facultative Wheat Zone; III, Middle and Lower Yangtze Valleys Autumn-sown Spring Wheat Zone; IV, Southwestern Autumn-sown Spring Wheat Zone; V, Southern Autumn-sown Spring Wheat Zone; VI, Northeastern Spring Wheat Zone; VII, Northern Spring Wheat Zone; VIII, Northwestern Spring Wheat Zone; IX, Qinghai-Tibetan Plateau Spring-Winter Wheat Zone; X, Xinjiang Winter-Spring Wheat Zone.
