# Supplementary material for: Identification of TaPPH-7A haplotypes and development of a molecular marker associated with important agronomic traits in common wheat
Source: BMC Plant Biol. 2019 Jul 8;19:296. doi: 10.1186/s12870-019-1901-0 (PMC6615193; doi:10.1186/s12870-019-1901-0)
Supplement: Supplementary file 6 — Table S4. The information of the wheat diversity panel and their genotypes of TaPPH-7A (DOCX 28 kb) [file 12870_2019_1901_MOESM6_ESM.docx]

**Additional file 6: Table S4.** The information of the wheat diversity panel and their genotypes of *TaPPH-7A*

| Number | Accession | Origin | Allele |
| --- | --- | --- | --- |
| 1 | PANDAS | Italy | A |
| 2 | An85 Zhong124-1 | Beijing | G |
| 3 | Yanzhan 1 | Henan | G |
| 4 | Bawangbian | Hebei | A |
| 5 | Beijing 10 | Beijing | G |
| 6 | Beijing 14 | Beijing | A |
| 7 | Cangzhouxiaomai | Hebei | A |
| 8 | Changwu 131 | Shaanxi | G |
| 9 | Chang 6878 | Shanxi | A |
| 10 | Dali 1 | Shaanxi | G |
| 11 | Dan R8093 | Beijing | G |
| 12 | Fengkang 13 | Beijing | A |
| 13 | Jimai 41 | Hebei | A |
| 14 | Jimai 6 | Hebei | A |
| 15 | Jin 2148-7 | Fujian | A |
| 16 | Jinghe 8922 | Beijing | A |
| 17 | Linkang 5108 | Shanxi | G |
| 18 | Baiqimai | Gansu | A |
| 19 | Changle 5 | Shandong | A |
| 20 | Hongheshang | Shanxi | A |
| 21 | Beijing 8686 | Beijing | A |
| 22 | 04-044 | Beijing | A |
| 23 | 04-030 | Beijing | G |
| 24 | Chun 22 9th-25 | CIMMITY | A |
| 25 | Ziganbaimangxian | Henan | G |
| 26 | Jingpin 10 | Beijing | G |
| 27 | Chun 04 9th-5-1 | CIMMITY | G |
| 28 | Chun 45 9th-50-1 | CIMMITY | G |
| 29 | Neixiang 188 | Henan | G |
| 30 | Jing 411 | Beijing | A |
| 31 | Chinese Spring | Sichuan | G |
| 32 | Baicaomai | Henan | G |
| 33 | Hanxuan 10 | Shanxi | G |
| 34 | Lumai 14 | Shandong | A |
| 35 | Opata | CIMMITY | A |
| 36 | W7984 | CIMMITY | A |
