# Supplementary material for: Identification of TaPPH-7A haplotypes and development of a molecular marker associated with important agronomic traits in common wheat
Source: BMC Plant Biol. 2019 Jul 8;19:296. doi: 10.1186/s12870-019-1901-0 (PMC6615193; doi:10.1186/s12870-019-1901-0)
Supplement: Supplementary file 7 — Table S5. Statistical analysis on traits of Population 1 (DOCX 28 kb) [file 12870_2019_1901_MOESM7_ESM.docx]

**Additional file 7: Table S5.** Statistical analysis on traits of Population 1

| Trait | Environment | Mean ± *SD* | Range | *CV* (%) |
| --- | --- | --- | --- | --- |
| SN | E1-E12 | 7.45±0.97 | 4.90-11.08 | 13.06 |
| GN | E1-E12 | 44.99±4.81 | 29.44-58.67 | 10.69 |
| TGW (g) | E1-E12 | 41.32±4.31 | 28.31-51.99 | 10.42 |
| Chl | E11-F | 56.27±3.64 | 46.63-67 | 6.47 |
|  | E11-GF | 52.57±8.13 | 7.6-64.3 | 15.46 |
|  | E12-F | 59.07±3.77 | 50.27-70.40 | 6.39 |
|  | E12-GF | 53.84±8.32 | 33.97-66.80 | 8.81 |

SN, spike number per plant; GN, grain number per spike; TGW, thousand-grain weight; Chl, Chlorophyll content; E1 to E12 indicate the environments of 2015-SY-WW, 2015-SY-DS, 2015-SY-WW+HS, 2015-SY-DS+HS, 2016-CP-WW,
